# Supplementary material for: Effects of Gestational Sleep Patterns and Their Changes on Maternal Glycemia and Offspring Physical Growth in Early Life
Source: Nutrients. 2022 Aug 18;14(16):3390. doi: 10.3390/nu14163390 (PMC9416776; doi:10.3390/nu14163390)
Supplement: Supplementary file 1 [file nutrients-14-03390-s001.zip › nutrients-1870686-supplementary.pdf]

Supplementary Online Content

Table S1. Comparisons of main characteristics of participants between the excluded group and the included group.
Table S2. Sensitivity analysis of effect of the maternal sleep pattern in early pregnancy and its change during pregnancy on physical growth of offspring within 24 months in the GDM subgroup [β/OR (95% CI)]. (Preterm birth excluded)
Figure S1. Sensitivity analysis of association between maternal night-time sleep duration in early pregnancy and offspring growth in early life [OR (95% CI)] (Preterm birth excluded)

Comparing the demographics of 3329 recruited eligible Maternal-Child pairs with the excluded 3385 participants, there was a significant difference between the FPG in middle pregnancy, education level and complications of the two groups and the results of included group were higher, while gestational weeks at delivery in the included group was relatively lower. No significant differences were observed in other variables between the two groups. (Table S1 below).

Table S1. Comparisons of main characteristics of participants between the excluded group and the included group.

Variables	Participants, N (%)		
	**Excluded (N=3385)**	**Included (N=3329)**	***P* value**
**Parents characteristics**			
Maternal age, mean (SD)	28.68 (4.20)	28.83 (4.10)	.14
Gestational weight gain, mean (SD), (Kg)	14.92 (5.44)	14.77 (5.18)	.27
FPG in middle pregnancy, mean (SD), (mmol/L)	4.43 (0.44)	4.48 (0.43)	**.003**
1h plasma glucose level, mean (SD), (mmol/L)	7.47 (1.69)	7.61 (1.79)	.10
2h plasma glucose level, mean (SD), (mmol/L)	6.59 (1.42)	6.61 (1.37)	.72
Pre-pregnancy BMI (Kg/m^2)			.01
18.5~23.9	2280 (67.36)	2237 (67.20)	
<18.5	610 (18.02)	532 (15.98)	
≥24.0	495 (14.62)	560 (16.82)	
Education level>12 (years)	2932 (86.62)	2995 (89.97)	**<.001**
Total family income ≤ ¥ 200 thousand (RMB)	2463 (72.76)	2442 (73.36)	.58
Parity-primiparous	1941 (57.34)	1878 (56.41)	.44
Depression in late pregnancy	385 (11.37)	409 (12.29)	.25
Anxiety in late pregnancy	373 (11.02)	393 (11.81)	.31
Energy intake in late pregnancy, mean (SD), (kcal)	2182.61 (1162.08)	2198.42 (1161.20)	.58
Complications	483 (14.27)	910 (27.34)	**<.001**
PA level in late pregnancy-low	1334 (39.41)	1338 (40.19)	**.03**
Father's age, mean (SD)	29.90 (4.78)	29.89 (4.69)	.95
Father's BMI, mean (SD), (kg/m2)	23.69 (3.47)	23.86 (3.48)	.05
**Offspring characteristics**			
Sex-male	1632 (48.21)	1648 (49.50)	.29
Gestational weeks at delivery, mean (SD),	39.03 (1.35)	39.10 (1.31)	**.03**
Delivery mode-Cesarean section	1698 (50.16)	1726 (51.85)	.17
Feeding practice within the-first-6-months			.94
Exclusive breastfeeding	1508 (44.55)	1474 (44.28)	
Mixed feeding	1077 (31.82)	1073 (32.23)	
Bottle-Feeding	800 (23.63)	782 (23.49)	
Breastfeeding duration, mean (SD), (months)	9.68 (5.03)	9.71 (5.08)	.82

Data are shown as N (%) unless otherwise indicated. Based on $\chi 2$ test, with Fisher exact test used for variables with any cell count< 10, or Kruskal-Walls test for continuous variables, $P<0.05$.

Sensitivity analysis results:

The sensitivity analyses did not materially alter the primary results. Compared to pregnant women with 7.5–8.5 hours of sleep in early pregnancy, those with < 7.5 hours of sleep or ≥ 8.5 hours of sleep had an increased risk of overweight/obesity in offspring within 24 months by 1.16 and 1.50 times, respectively (OR=2.16; 95% CI, 1.07 to 4.36; OR=2.50; 95% CI, 1.11 to 5.65). Always poor sleep quality from early to late pregnancy was associated with a 32% increased risk of offspring overweight/obesity (OR=1.32; 95% CI, 1.04 to 1.66), while increased sleep duration from early to late pregnancy significantly reduced the risk of overweight/obesity by 67% (OR=0.33; 95% CI, 0.14 to 0.79). For pregnant women in the non-GDM group, a change from poor to good sleep quality from early to late pregnancy was a protective factor for the occurrence of catch-up growth in offspring, reducing the risk by 15% (Table S2). The Figure S1 still showed a U-shaped curve relationship between maternal sleep duration in early pregnancy and offspring overweight/obesity in early life, with almost 8 hours as the node.

Table S2. Sensitivity analysis of effect of the maternal sleep pattern in early pregnancy and its change during pregnancy on physical growth of offspring within 24 months in the GDM subgroup [β/OR (95% CI)]. (Preterm birth excluded)

	Non-GDM			GDM		
	BAZ[a]	Catch-up growth[b]	Overweight/Obesity[b]	BAZ[a]	Catch-up growth[b]	Overweight/Obesity[b]
**Sleep level in early-pregnancy**						
Total PSQI score	0.01 (-0.00, 0.01)	1.01 (0.99, 1.03)	1.01 (0.99, 1.03)	0.01 (-0.00, 0.02)	0.98 (0.95, 1.01)	1.02 (0.99, 1.05)
Sleep quality						
Good	Ref.	Ref.	Ref.	Ref.	Ref.	Ref.
Poor	-0.02 (-0.06, 0.02)	1.04 (0.95, 1.14)	0.98 (0.89, 1.08)	0.04 (-0.03, 0.10)	1.10 (0.92, 1.31)	1.10 (0.92, 1.31)
Sleep duration-continuous (hour)	-0.06 (-0.17, 0.06)	0.78 (0.55, 1.10)	1.01 (0.77, 1.34)	-0.04 (-0.20, 0.12)	0.68 (0.40, 1.18)	0.91 (0.63, 1.32)
Sleep duration- categorical (hour)						
7.5~8.5	Ref.	Ref.	Ref.	Ref.	Ref.	Ref.
<7.5	0.12 (-0.07, 0.31)	1.49 (0.87, 2.53)	1.35 (0.84, 2.16)	0.15 (-0.15, 0.44)	2.97 (0.90, 9.80)	**2.16 (1.07, 4.36)**
≥8.5	0.08 (-0.17, 0.34)	1.02 (0.54, 1.95)	1.52 (0.80, 2.89)	0.17 (-0.26, 0.59)	2.18 (0.52, 9.07)	**2.50 (1.11, 5.65)**
**Changes in sleep quality during pregnancy**						
Sleep quality						
Always good	Ref.	Ref.	Ref.	Ref.	Ref.	Ref.
Always poor	-0.01 (-0.06, 0.04)	0.98 (0.87, 1.11)	0.98 (0.86, 1.12)	0.05 (-0.03, 0.14)	1.16 (0.92, 1.47)	**1.32 (1.04, 1.66)**
From good to poor	-0.00 (-0.06, 0.05)	**0.83 (0.73, 0.95)**	0.99 (0.87, 1.14)	0.03 (-0.07, 0.12)	1.19 (0.93, 1.53)	1.18 (0.91, 1.53)
From poor to good	-0.05 (-0.11, 0.02)	**0.85 (0.72, 0.98)**	0.95 (0.80, 1.13)	0.04 (-0.06, 0.14)	1.24 (0.96, 1.62)	1.26 (0.96, 1.66)
Changes in sleep duration during pregnancy						
Shortened sleep duration	Ref.	Ref.	Ref.	Ref.	Ref.	Ref.
Prolonged sleep duration	0.10 (-0.10, 0.31)	0.83 (0.46, 1.48)	1.19 (0.72, 1.98)	-0.34 (-0.72, 0.03)	1.47 (0.33, 6.46)	**0.33 (0.14, 0.79)**

[a] β (95% CI), [b] OR (95% CI). Adjusted for education, income, maternal age, history of diabetes, pre-pregnancy BMI, gestational weight gain, complications, parity, gestational age, delivery mode, sex, maternal smoking or drinking, depression, anxiety, PA level and energy intake in late-pregnancy, napping in early and late pregnancy, gestational weeks at delivery, father's BMI, sex, feeding practice within the-first-6-months and breastfeeding duration.

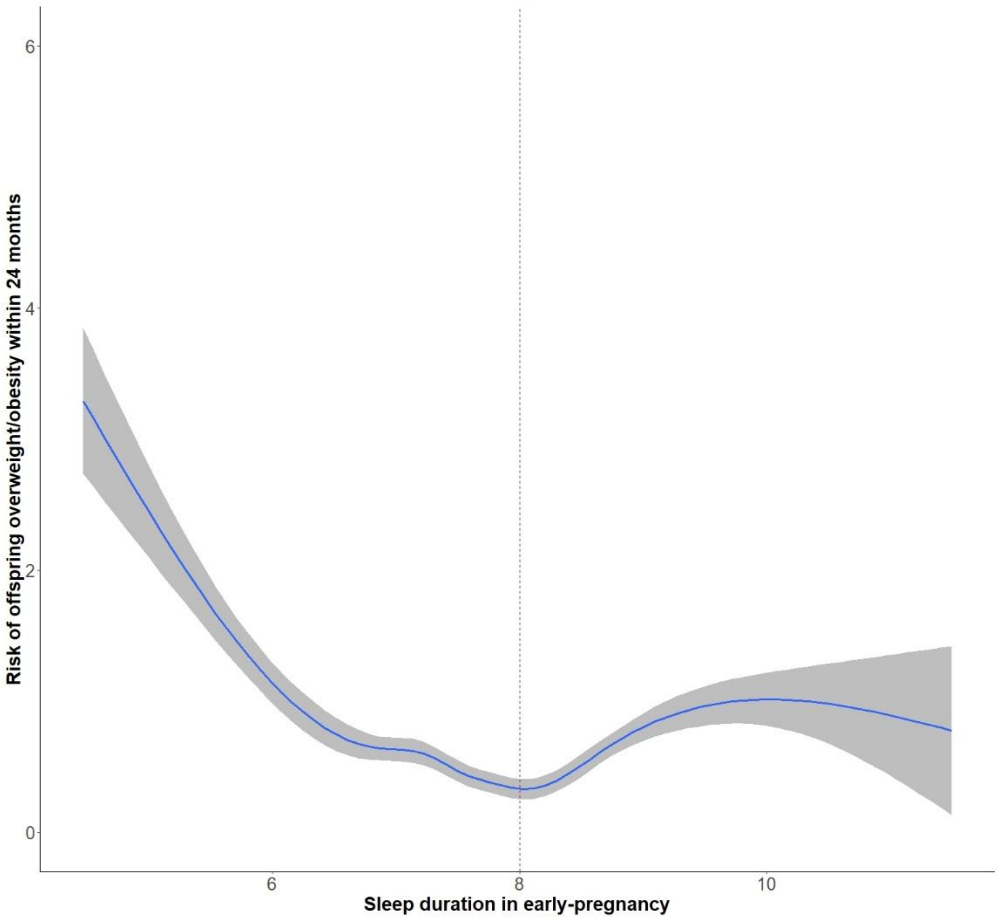

Figure S1. Sensitivity analysis of association between maternal night-time sleep duration in early pregnancy and offspring growth in early life [OR (95% CI)] (Preterm birth excluded)

Adjusted for education, income, maternal age, history of diabetes, pre-pregnancy BMI, gestational weight gain, complications, parity, gestational age, delivery mode, sex, maternal smoking or drinking, depression, anxiety, PA level and energy intake in late-pregnancy, napping in early and late pregnancy, gestational weeks at delivery, father's BMI, sex, feeding practice within the-first-6-months and breastfeeding duration.
